# Impact of Somatic Mutations in the D-Loop of Mitochondrial DNA on the Survival of Oral Squamous Cell Carcinoma Patients

**DOI:** 10.1371/journal.pone.0124322

**Published:** 2015-04-23

**Authors:** Jin-Ching Lin, Chen-Chi Wang, Rong-San Jiang, Wen-Yi Wang, Shih-An Liu

**Affiliations:** 1 Department of Radiation Oncology, Taichung Veterans General Hospital, Taichung, Taiwan; 2 Department of Otolaryngology, Taichung Veterans General Hospital, Taichung, Taiwan; 3 Faculty of Medicine, School of Medicine, National Yang-Ming University, Taipei, Taiwan; 4 Department of Nursing, Hung-Kuang University, Taichung, Taiwan; School of Medicine, Fu Jen Catholic University, TAIWAN

## Abstract

**Objectives:**

The aim of this study was to investigate somatic mutations in the D-loop of mitochondrial DNA (mtDNA) and their impact on survival in oral squamous cell carcinoma patients.

**Materials and Methods:**

Surgical specimen confirmed by pathological examination and corresponding non-cancerous tissues were collected from 120 oral squamous cell carcinoma patients. The sequence in the D-loop of mtDNA from non-cancerous tissues was compared with that from paired cancer samples and any sequence differences were recognized as somatic mutations.

**Results:**

Somatic mutations in the D-loop of mtDNA were identified in 75 (62.5%) oral squamous cell carcinoma patients and most of them occurred in the poly-C tract. Although there were no significant differences in demographic and tumor-related features between participants with and without somatic mutation, the mutation group had a better survival rate (5 year disease-specific survival rate: 64.0% vs. 43.0%, *P* = 0.0266).

**Conclusion:**

Somatic mutation in D-loop of mtDNA was associated with a better survival in oral squamous cell carcinoma patients.

## Introduction

Oral cancer was reported to be the sixth most common cancer in the world with an estimated 400,000 newly diagnosed cases and 223,000 mortalities in 2008 [1,2]. The incidence is higher in developing countries. Since 1991, oral cancer has been one of the top 10 causes of death from cancer in Taiwan. According to the statistical data from Taiwan’s Ministry of Health and Welfare, the annual death toll for oral cancer in men has been climbing rapidly [3]. No promising progress in the treatment of oral squamous cell carcinoma has been made over the past decade. Although better combinations of loco-regional therapeutic protocols, such as radical extirpation plus radiation after neoadjuvant chemotherapy or bioradiation, have enhanced the quality of life after diagnosis, the 5-year survival rate has not changed much in recent years [4]. Therefore, the ability to identify poor prognosticators has become an important issue in clinical practice.

Mitochondrion provides cells with energy and plays a crucial role in the launch and execution of apoptosis. Apart from the nucleus, it is the only other organelles in mammalian cells to bear a genome. Human mitochondrial DNA (mtDNA) is a 16.5-kb circular double-stranded DNA molecule which has a high number of copies per cell. It contains genes coding for 13 peptides engaged in respiration and oxidative phosphorylation, 2 rRNAs, and a set of 22 tRNAs that are essential for protein synthesis in mitochondria [4]. Furthermore, mtDNA encloses a non-coding area comprising an exclusive displacement loop (D-loop) that handles the replication and transcription of mtDNA. It has been acknowledged that the mitochondrial genome is highly susceptible to oxidative damage and mutation mainly due to the high rate of reactive oxygen species generation and the ineffective DNA repair system in the organelle [5]. As a result, mtDNA is more vulnerable than nuclear DNA in cancer cells. It is estimated that mtDNA has a 10-fold higher rate of accumulation of mutations than that of nuclear DNA.

Somatic mutations of mtDNA have been identified in many human cancers, such as colorectal cancer, breast cancer, hepatocellular carcinoma, and gastric carcinoma [6–8]. Mutations have been detected in the protein coding genes, rRNA genes and in the D-loop region. Previous studies have noted that 56.7% to 66.7% of the oral squamous cell carcinoma patients had somatic mutations in the D-loop of mtDNA [9,10]. However, few studies have discussed the association between somatic mutations of the D-loop of mtDNA and survival of oral squamous cell carcinoma patients. Therefore, this study investigated somatic mutations in the D-loop of mtDNA and examined its impact on survival of oral squamous cell carcinoma patients.

## Materials and Methods

This study was approved by the Institutional Review Board of Taichung Veterans General Hospital. Participants who were scheduled to receive surgical resection of oral cancer from August 2008 to June 2011 were eligible for enrollment in this study. The entire protocol was explained in detail to all participants and written consent was acquired prior to enrollment. Patients who refused surgery, had a histological type other than squamous cell carcinoma, had insufficient chart records, or declined to participate in this study were excluded. Basic demographic data including age, gender, primary tumor site, pathological stage, and histological characteristics were recorded. Therapeutic plans for all participants were all in accordance to the consensus guidelines of the head and neck cancer team of our hospital. Moreover, all surgical procedures were carried out by a single surgeon (SA Liu). Mutation analysis was performed blindly by the staff of laboratory.

### Human oral squamous cell carcinoma tissues, peripheral blood, and DNA extraction

Histologically confirmed oral squamous cell carcinoma and corresponding non-cancerous mucosa tissues were obtained from participants. All samples were placed in liquid nitrogen immediately after microdissection. Peripheral blood (10 ml) was also drawn before operation and was injected into an EDTA-treated tube. Then the sample was centrifuged at 1000×g for 15 minutes, and the plasma was transferred to 1.5-ml microtubes. The peripheral blood mononuclear cell layer was relocated into a clean 50 ml centrifuge tube, washed twice with a balanced salt solution, and centrifuged at 150×g for 10 minutes. The samples were stored at -30°C until use. Total DNA was extracted using the QIAamp DNA Mini kit (QIAGEN) according to the manufacturer’s instructions. The final DNA was dissolved in doubly distilled water and frozen at -30°C until further processing.

### Direct sequencing of the D-loop region of mtDNA

The D-loop region of mtDNA was analyzed for mutations using direct sequencing of the products from polymerase chain reaction (PCR). The primer pairs L16190 (mucleotide position (np) 16190–16209, 5’-CCCCATGCTTACAAGCAAGT-3’) and H602 (np 602–583, 5’-GCTTTGAGGAGGTAAGCTAC-3’) were utilized for amplification of a 982 bp DNA fragment from the D-loop region of mtDNA. All the PCR products were purified and sequenced with an ABI Big Dye Terminator (version 3.1) cycle sequencing ready reaction kit and an ABI PRISM 3100 sequencer (Applied Biosystems, Foster City, CA). The D-loop of mtDNA sequence from non-tumor oral mucosa and tumor specimens of the same participant were analyzed and any DNA sequence differences were regarded as somatic mutations.

### Statistical Analysis

Demographic data was presented as descriptive statistics. Moreover, comparison of continuous variables between participants with and without somatic mutations of the D-loop of mtDNA were analyzed by Student’s *t* test, whereas nominal or ordinal variables were analyzed using the Chi-square test or Fisher’s exact test. Survival analysis was calculated by the Kaplan-Meier method and the differences among subgroups were examined by the log-rank test. All analyses were computed by SPSS for Windows, version 12.1 (SPSS, Chicago, IL) and a *p* < 0.05 was considered statistically significant.

## Results

A total of 129 participants scheduled to undergo surgical resection for oral cancer were eligible for enrollment in this study. Among them, 4 participants declined to participate in the study. In addition, 3 participants refused surgery and received organ preservation treatment instead, and 2 participants had a histological type of cancer other than squamous cell carcinoma. Adequate data were obtained from 120 participants. The average age of the participants was 52.1 ± 9.3 years and males accounted for 90.0% (n = 108) of all participants. Near half of the participants had primary site at the tongue (n = 48, 40.0%), followed by buccal mucosa (n = 33, 27.5%), tonsil (n = 23, 19.2%) and soft palate (n = 8, 6.7%). In terms of personal habits, 96 participants (80.0%) were smokers, 84 (70.0%) consumed alcohol regularly, and 76 (63.3%) habitually chewed betel quid. Sixteen participants (13.3%) had stage I diseases, whereas 20 (16.7%), 12 (10.0%), and 72 (60.0%) participants had stage II, III, and IV diseases, respectively. Fifty participants (41.7%) died and 66 participants (55.0%) were disease-free during the follow-up period. The average follow-up duration was 34.2 months (± 23.6 months).

By direct sequencing, 62.5% (75 out of 120) of the oral squamous cell carcinoma samples carried somatic mutations in the D-loop of mtDNA. Most of the mtDNA mutations in oral squamous cell carcinoma were heteroplasmic (48/75, 64.0%). Sixty-four oral squamous cell carcinomas (85.3%) showed alterations in the mononucleotide repeat situated in the polycytidine stretch over np 303 of mtDNA. Details of the somatic mutations in the D-loop region of mtDNA are shown in Table 1.

**Table 1:** Somatic mutations in the D-loop region of mtDNA of oral cancers.

Patient code	Nucleotide position	Somatic mutation	Homoplasmy
01	303	C8 → C8-9	Heteroplasmy
02	303	C8-9 → C8-10	Heteroplasmy
05	317	C5 → C6	Yes
07	303	C7 → C7-8	Heteroplasmy
	16304	C/T → C	
09	303	C8-9 → C8-10	Yes
11	303	C7 → C7-9	Heteroplasmy
12	303	C7-8 → C8-9	Heteroplasmy
15	303	C8 → C9	Yes
	16257, 16316	T → C	
16	205	T/C → T	Yes
	312	C8 → C9	
17	312	C8-9 → C8	Yes
	16340	A → G	
	16524	T → C	
21	303	C8 → C8-9	Heteroplasmy
25	303	C15 → C15-18	Heteroplasmy
26	312	C8 → C7-9	Heteroplasmy
28	303	C9 → C8	Yes
31	303	C8-10 → C9-10	Yes
34	369, 414, 467	G → A	Heteroplasmy
	414	C → G/C	
35	312	C8 → C9	Yes
36	303	C7 → C7-8	Heteroplasmy
38	303	C7-9 → C7-10	Yes
39	303	C7 → C7-8	Heteroplasmy
40	2288	G → G/T	Heteroplasmy
41	303	C8 → C8-9	Heteroplasmy
42	303	C8 → C7	Yes
44	303	C8 → C8-9	Heteroplasmy
45	303	C8 → C8-9	Heteroplasmy
46	303	C8-9 → C8-10	Heteroplasmy
47	303	C8 → C8-9	Heteroplasmy
49	303	C7-8 → C8-9	Yes
53	146, 150	C → T	Heteroplasmy
	199, 16261	T → C/T	
55	303	C8-10 → C8	Yes
56	303	C8 → C8-9	Heteroplasmy
57	303	C8-9 → C8-10	Heteroplasmy
58	369, 392, 567	G/A → A/G	Heteroplasmy
59	66	Deletion G	Heteroplasmy
	369	G/A → A/G	
60	303	C7 → C7-8	Heteroplasmy
61	303	C8 → C8-9	Heteroplasmy
	66	G6 → G5-6	
62	303	C8 → C8-9	Heteroplasmy
63	303	C8 → C8-9	Heteroplasmy
64	303	C8-9 → C8	Yes
	13135	G → A	
	13152	A → G	
65	303	C8 → C8-9	Heteroplasmy
	10230	G → G/A	
67	303	C8-9 → C8-10	Yes
72	303	C8-9 → C8-10	Heteroplasmy
73	303	C8 → C8-9	Heteroplasmy
75	303	C8-9 → C8	Yes
	9053	G → A	
	10310	G → A	
76	303	C8 → C8-9	Heteroplasmy
79	303	C8-9 → C8	Yes
	9824	T → C	
80	303	C8 → C8-9	Heteroplasmy
	3882	A → A/G	
	6392	T → T/C	
	10398	A → A/G	
83	303	C8-9 → C8	Yes
84	3882	A/G → G	Yes
	3970	C/T → C	
88	303	C8 → C8-9	Heteroplasmy
93	303	C8 → C8-9	Heteroplasmy
94	303	C8-9 → C8	Yes
95	303	C8 → C8-9	Heteroplasmy
97	303	C8-9 → C8	Yes
98	303	C7-8 → C7	Yes
100	303	C8 → C8-9	Heteroplasmy
101	303	C8-10 → C8-9	Heteroplasmy
103	303	C8 → C8-10	Heteroplasmy
	514	CA5 → CA4-5	
105	303	C8 → C8-9	Heteroplasmy
106	303	C8-10 → C9-10	Yes
109	303	C8-10 → C9-10	Yes
110	303	C8-10 → C8-10	Heteroplasmy
111	303	C8-9 → C8-10	Heteroplasmy
113	303	C8-9 → C8-9	Heteroplasmy
	514	CA5 → CA4-5	
114	303	C8-9 → C8-9	Heteroplasmy
115	303	C8-9 → C8-9	Heteroplasmy
	514	CA5 → CA4-5	
118	303	C8-9 → C8	Yes
121	303	C8-10 → C8-9	Heteroplasmy
122	303	C8-9 → C8-10	Heteroplasmy
123	303	C8-10 → C8-9	Yes
124	303	C8-9 → C8-10	Heteroplasmy
125	66	G6 → G5-6	Heteroplasmy
	303	C8-9 → C9-10	
126	303	C8 → C8-9	Yes
127	66	G6 → G5-6	Heteroplasmy
	303	C7 → C7-8	
129	303	C8 → C8-9	Yes

Abbreviation: mtDNA → mitochondrial DNA

**Fig 1:**
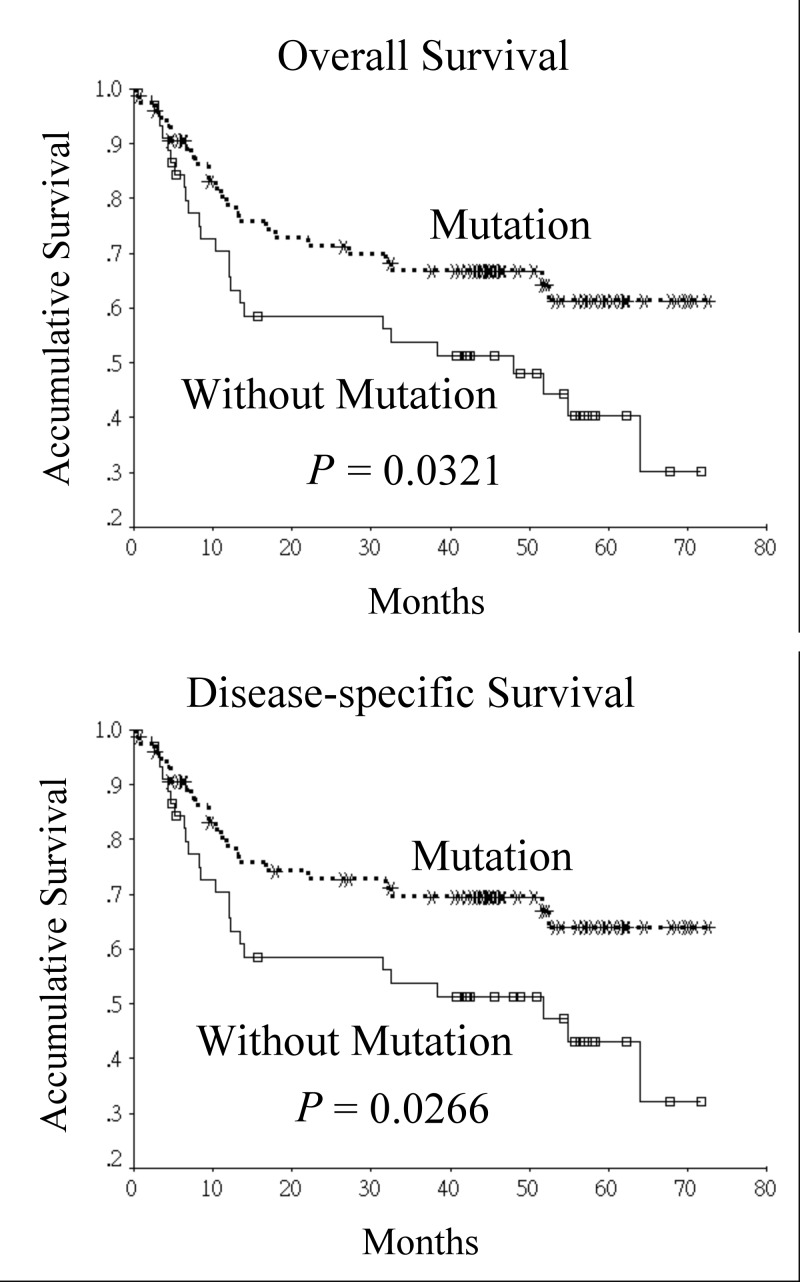
Univariate analysis of the effect of somatic mutations in the D-loop of mitochondrial DNA on survival of oral squamous cell carcinoma patients.

**Fig 2:**
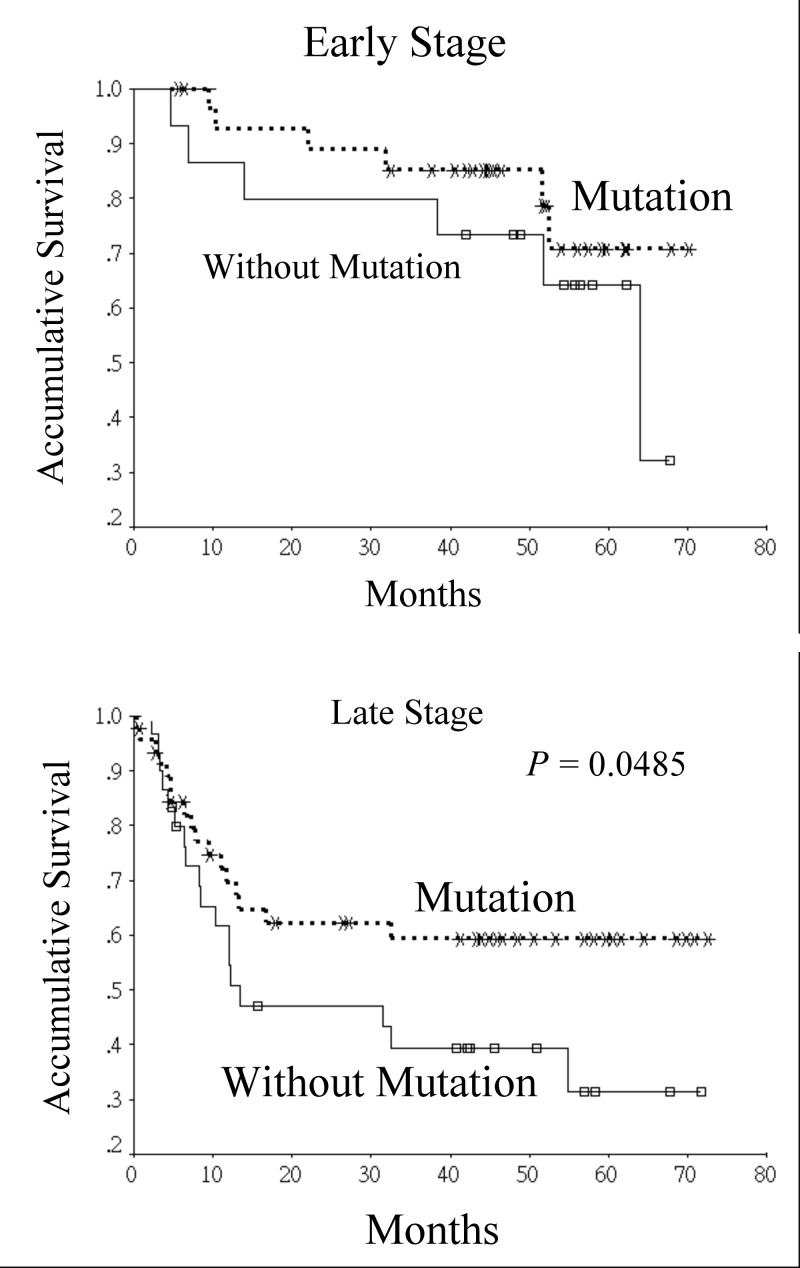
Disease-specific survival curves of oral squamous cell carcinoma patients based on the somatic mutations in the D-loop of mitochondrial DNA and stratified by pathological stage.

The participants were then separated into 2 groups based on the status of somatic mutations in the D-loop of mtDNA. Therefore, 75 participants were in the mutation group while 45 participants were in the non-mutation group. Comparisons of variables between the 2 groups are shown in Table 2. No significant difference were noted in the distribution of age between the 2 groups (mutation group vs. non-mutation group: 51.5 ± 9.5 vs. 53.1 ± 9.1 years, *P* = 0.365). In addition, there were no significant differences between the 2 groups in gender, lifestyle habits, histological characteristics, peri-neural invasion, angiolymphatic invasion, pathological stage, postoperative radiotherapy, and presence of recurrence or distant metastasis. Although participants without somatic mutation of D-loop of mtDNA were more likely to have extranodal spread in metastatic cervical lymph nodes, no significant statistical differences were noted (24.4% vs. 10.7%, *P* = 0.081). In participants with smoking habit, there was no significant difference in the amount of smoking between two groups (35.3 pack-year vs. 34.5 pack-year, *P* = 0.866). However, in habitual betel quid chewers, those with somatic mutation of D-loop of mtDNA tended to chew more betel quid when compared with those without (419 piece-year vs. 276 piece-year, *P* = 0.025). According to the Kaplan-Meier survival analysis, the prognosis of participants with somatic mutations of the D-loop of mtDNA was better than that of those without (5-year overall survival and disease-specific survival: 61.4% vs. 40.3%, *P* = 0.0321, 64.0% vs. 43.0%, *P* = 0.0266, respectively) (Fig 1). A significant difference in disease-specific survival was still noted after stratifying the participants according to their pathological stage. (early stage: 70.8% vs. 64.2%, late stage: 59.4% vs. 31.5%, *P* = 0.0485) (Fig 2).

**Table 2:** Descriptive and bivariate analysis of oral squamous cell carcinoma patients with or without somatic mutations of D-loop of mitochondrial DNA.

Variables	Total no. of patients (% in column)	No. of patients (%)		*P* value
		Mutation group (n = 75)	Non-mutation group (n = 45)	
**Age (yr)**				0.961
< 49 years	47(39.2%)	30(63.8%)	17(36.2%)	
> = 50 years	73(60.8%)	45(61.6%)	28(38.4%)	
**Gender**				0.999 †
Female	12(10.0%)	8(66.7%)	4(33.3%)	
Male	108(90.0%)	67(62.0%)	41(38.0%)	
**Personal Habit**					
Smoking	96(80.0%)	59(61.5%)	37(38.5%)	0.814
Alcohol	84(70.0%)	51(60.7%)	33(39.3%)	0.681
Betel quid	76(63.3%)	51(67.1%)	25(32.9%)	0.240
**Primary tumor sites**				0.157
Lip	2(1.7%)	2(100.0%)	0(0%)	
Gum	2(1.7%)	1(50.0%)	1(50.0%)	
Floor of mouth	3(2.5%)	2(66.7%)	1(33.3%)	
Tongue	48(40.0%)	35(72.9%)	13(27.1%)	
Buccal	33(27.5%)	22(66.7%)	11(33.3%)	
Palate	8(6.7%)	4(50.0%)	4(50.0%)	
Retromolar trigone	1(0.8%)	1(100.0%)	0(0%)	
Tonsil	23(19.2%)	8(34.8%)	15(65.2%)	
**Histological features**				0.307
Well differentiated	20(16.7%)	13(65.0%)	7(35.0%)	
Moderately differentiated	62(51.7%)	42(67.7%)	20(32.3%)	
Poorly or undifferentiated	38(31.7%)	20(52.6%)	18(47.4%)	
**Perineural invasion**				0.917
Yes	30(25.0%)	19(63.3%)	11(36.7%)	
No	90(75.0%)	56(62.2%)	34(37.8%)	
**Angiolymphatic invasion**				0.231
Yes	30(25.0%)	16(53.3%)	14(46.7%)	
No	90(75.0%)	59(65.6%)	31(34.4%)	
**Extranodal spread**				0.081
Yes	19(15.8%)	8(42.1%)	11(57.9%)	
No	101(84.2%)	67(66.3%)	34(33.7%)	
**Pathological stage**				0.696
Stage I-II	36(30.0%)	23(63.9%)	13(36.1%)	
Stage III-IV	84(70.0%)	51(60.7%)	33(39.3%)	
**Postoperative radiotherapy**				0.952
Yes	97(80.8%)	60(61.9%)	37(38.1%)	
No	23(19.2%)	15(65.2%)	8(34.8%)	
**Recurrence/distant metastasis**				0.394
Yes	54(45.0%)	31(57.4%)	23(42.6%)	
No	66(55.0%)	44(66.7%)	22(33.3%)	

^†^ Fisher’s exact test

Somatic mutation of D-loop of mtDNA in paired plasma or blood was also examined and compared with that of tumor tissues in participants with somatic mutation of the D-loop of mtDNA. Our data showed that 45 plasma samples (60.0%) had somatic mutation of the D-loop of mtDNA whereas 47 blood samples (62.7%) had somatic mutation of the D-loop of mtDNA. When comparing somatic mutation of the D-loop of mtDNA between plasma and tumor specimens, the pattern was similar in 12 pairs of samples (26.7%). In blood, the pattern was comparable in 11 pairs of samples (23.4%).

## Discussion

In the current study, we found more than half of the oral squamous cell carcinoma samples carried somatic mutation in the D-loop region of mtDNA and most of them were heteroplasmic. The D-loop region of mtDNA is a crucial position for replication and expression of the mitochondrial genome because it possesses essential transcriptional promoters and is the leading-strand origin of replication [11]. Furthermore, the D-loop region is hypervariable and susceptible to somatic mutations because of its distinctive triple-stranded DNA structure. Mitochondrial mutations may modify the function of normal oxidative phosphorylation chain which operates as a metabolic caretaker to prevent unexpected alterations to the glycolytic metabolic phenotype and also serves as a gatekeeper to avoid improper production of genotoxic reactive oxygen species [12]. Heteroplasmy have been reported previously in mitochondrial diseases at specific mitochondrial genes as homoplasmic mutations would be lethal [13]. Previous studies found a lower mutation rate of mtDNA ranged from 21% to 28.9% in head and neck tumor samples [12,14]. The discrepancy might be due to the diverse studied populations. In the current study, all participants had oral squamous cell carcinoma while the abovementioned studies included a variety of head and neck cancer patients. Tan et al. in their study of betel quid-related oral cancer patients found that the somatic mutation of D-loop region developed in 66.7% (12/18) of samples [9]. A recent study on oral squamous cell carcinoma also found a high rate of somatic mutations in the D-loop region (203 out of 240, 85.0%) [15]. The high somatic mutation rates in the aforementioned studies were comparable with the rate in our study.

In the Kaplan-Meier survival analysis, our study found that oral squamous cell carcinoma patients with somatic mutation of the D-loop region had better prognosis when compared with those without. The survival advantage still existed after stratifying patients based on pathological stage. A previous study that investigated the relationships between clinico-pathological factors and mitochondrial D-loop mutations in head and neck carcinoma found that the presence of D-loop mutation was not associated with prognosis or with response to neoadjuvant chemotherapy. The 5-year overall survival of patients with somatic D-loop mutation was 81% compared to 70% in patients without (*p* = 0.71) [14]. However, abovementioned study included a variety of head and neck cancer patients whereas our study only included oral squamous cell carcinoma patients. Nevertheless, it seemed that patients with somatic D-loop mutation still had a higher survival rate. Lai et al. in their study on mutations in oral squamous cell carcinoma patients found that pathogenic mutations were associated with a poorer disease-free survival when compared with those without [15]. The difference might be explained by dissimilar methodological designs as the aforementioned study correlated pathological mutation with survival while our study focused on somatic mutation of the D-loop.

Although the differences between patients with somatic D-loop mutation and those without were not statistically significant in betel quid chewing, primary tumor sites, histological features, and recurrence/distant metastasis status, slight variation existed between the two groups. It is possible that non-mutation group might have a higher percentage of patients with mutations in other genes (such as p53, retinoblastoma [Rb], ataxia-telangiectasia mutated [ATM], etc.) when compared with that of mutation group. Loss of Rb function may result in uncontrolled proliferation of cells and down-regulation of Rb was associated with poorer disease-free survival in oral cancer patients [16]. In addition, ATM kinase plays a critical role in the DNA damage response and its phosphorylation cascade to inhibit the p53-MDM2 interaction, which releases p53 to induce p21 and G1 cell-cycle arrest [17]. All these factors might correlate with prognosis of oral cancer patients.

An earlier study suggested that the mutation of the D-loop region was an early event in head and neck carcinogenesis [18]. They found the D-loop mutation rate increased from 22% in premalignant lesions to 50% in lesions of severe dysplasia and even 61% in carcinomas *in situ*. Another study also suggested that mutations in mtDNA might be involved in the development of cancer [19]. Although mtDNA mutation could play a role in carcinogenesis, it may not necessarily influence the survival. For example, human papillomavirus (HPV) is closely associated with oropharyngeal cancer, yet patients with HPV-related oropharyngeal cancer have a better survival than those without HPV infection [20]. Lee et al. found a higher rate of mutations in the D-loop of mtDNA in human cancer with advanced stage [8]. The findings suggest the possibility that the alterations increased in cancer during its progression stage because of the highly unstable nature of mtDNA [8]. In a recent case control study from Northeast India, a significant correlation was also noted between the increased number of D-loop mutations with the advancement in patients’ tumor stage (*P* = 0.009, r = 0.48) [13]. However, our study failed to demonstrate such a correlation, probably due to the different race and environment in the present study compared with the aforementioned study. In addition, differences in the distribution of tumor sites among studies may explain such diverse results.

Our study showed that the most frequent mutation site in the D-loop of mtDNA originated in the polycytidine stretch. This phenomenon was also reported in previous studies [8–10,15]. The D-loop was reported to be highly vulnerable to oxidative damage when compared with the other regions of mtDNA [21]. The extensive oxidative damage to the polycytidine sequences may cause slipping and/or misincorporation during replication or repair of mtDNA by mitochondrial DNA polymerase. As a result, an increased rate of replicative errors was noted in two well-known mono-nucleotide polymorphism repeats located in the D-loop region [15].

Zhou et al. in their study of frequency and phenotypic implications of mtDNA mutations in head and neck squamous cell carcinoma found that the mutations of mtDNA correlated positively with p53 mutations [12]. However, as equivalent data were not collected in the current study, no comparison could be made. Other studies indicated that the mutations of the D-loop were correlated with tobacco consumption and betel quid chewing [9,14]. We found that oral squamous cell carcinoma patients with heavy betel quid consumption had an increased somatic mutation frequency in the D-loop of mtDNA. Reactive oxygen species generated by areca nut and lime have been shown to induce oxidative damage to DNA [22]. A previous study also indicated that betel quid chewing significantly induced the accumulation of mtDNA deletions in human oral tissues [23]. This might explain why heavy betel chewers were most likely to have a somatic mutation in the D-loop region of mtDNA in the current study.

In contrast to the findings of the present study, Zhang et al. showed that genetic polymorphisms in the D-loop are independent poor prognostic markers in patients with esophageal squamous cell carcinoma [24]. One reasons for the discrepancy in the aforementioned results might be that Zhang et al. investigated patients with esophageal cancer and analyzed peripheral blood rather than tumor specimens. In addition, the abovementioned study examined absolute mutation rather than somatic mutation. Furthermore, the frequency of single nucleotide polymorphisms in the study population was not provided. Challen et al. in their study of mtDNA mutations suggested that somatic mutations of the D-loop were uncommon and may not have an impact on the prognosis in head and neck cancer patients [25]. Nevertheless, the aforementioned study was conducted in a non-endemic betel quid chewing area. Moreover, the studied populations were somewhat different. The aforementioned study included a variety of head and neck cancers, such as oral cavity, hypopharynx, oropharynx, and larynx whereas our study only included oral squamous cell carcinoma patients. The reason why somatic mutation of D-loop of mtDNA influences the survival of oral cancer patients is unknown. Although our study found a better prognosis in patients with somatic mutation of the D-loop of mtDNA, further study is warranted to elucidate the exact mechanism.

Uzawa et al. showed that circulating mtDNA rather than genomic DNA might be a valuable blood test for evaluation of prognosis in cancer patients. Oral squamous cell carcinoma patients with recurrence were most likely to have higher amounts of mutant mtDNAs when compared with those without recurrence [26]. Conversely, we found only about one-fourth of patients carried the same pattern of somatic mutation of mtDNA in tumors and peripheral blood samples. Therefore, the prognostic value of somatic mutation of mtDNA in peripheral blood was doubtful in our study. A notable difference between the abovementioned study and ours was the timing of the blood sampling. The abovementioned study obtained a blood sample before and after treatment, while our study only obtained a blood sample before surgery. In addition, the aforementioned study used quantitative real-time PCR combined with high-resolution melting curve analysis, whereas our study used direct sequencing of the PCR products. Furthermore, they used the percentages of mutant mtDNA while we used the presence of somatic mutation of mtDNA.

There were some limitations in our study. First, the external validity of the findings is limited as it was conducted at a single hospital. Second, the power of our study is probably low due to the relatively small sample size. Third, we did not collect data about HPV status in specimens, which is a well-known prognostic factor for oropharyngeal cancer. Finally, although the treatment guidelines are standardized in our hospital, inevitably there were individual differences among patients.

In conclusion, a high rate of somatic mutations in the D-loop region of mtDNA was noted in our oral squamous cell carcinoma patients in a betel quid endemic region. Better overall and disease-specific survival rates were noted in patients with somatic mutations in the D-loop region when compared with those of patients without somatic mutation. The correlation between plasma/blood and tumor tissues in patients with somatic mutation of the D-loop of mtDNA was poor. Further study with a larger population is warranted in order to elucidate the relationship between the D-loop mutations and survival in oral squamous cell carcinoma patients.
